# Efficacy of Adalimumab in a Girl with Refractory Intestinal Behcet's Disease

**DOI:** 10.1155/2015/716138

**Published:** 2015-11-02

**Authors:** Mariko Kaji, Takayuki Kishi, Takako Miyamae, Satoru Nagata, Hisashi Yamanaka, Satoshi Fujikawa

**Affiliations:** ^1^Department of Pediatrics, Tokyo Women's Medical University, No. 8-1, Kawadacho, Shinjuku-ku, Tokyo 162-0054, Japan; ^2^Institute of Rheumatology, Tokyo Women's Medical University, No. 10-22, Kawadacho, Shinjuku-ku, Tokyo 162-0054, Japan; ^3^Fujikawa Pediatrics Clinic, No. 7-29-2, Ooi, Shinagawa-ku, Tokyo 140-0014, Japan

## Abstract

We describe our experience with a juvenile patient who had refractory intestinal Behcet's disease that responded to adalimumab, a fully humanized antibody against soluble TNF-**α** and its receptor. The patient, a 13-year-old girl, presented with oral aphthous ulcers, vulvar pain, and rashes on the lower extremities. She gradually developed a low-grade fever, abdominal pain, diarrhea, and hematochezia. Lower gastrointestinal endoscopy revealed ulcers in the terminal ileum, consistent with intestinal Behcet's disease. Methylprednisolone pulse therapy was initiated, after which the symptoms transiently improved, but, during the corticosteroid taper, the abdominal pain recurred. The symptoms resolved soon after the administration of adalimumab. Of importance, the dose of corticosteroids was successfully reduced without exacerbation during 8 months of observation. This is the first reported case in which adalimumab was used for pediatric gastrointestinal Behcet's disease. Adalimumab is a good choice for intestinal Behcet's disease refractory to conventional treatment.

## 1. Introduction

Behcet's disease is a chronic relapsing vasculitis that is characterized by recurrent oral aphthous and genital ulcers with uveitis [1]. In addition to the main symptoms, there are some much less common manifestations, such as gastrointestinal or central nervous system disease. Pediatric Behcet's disease occurs in approximately 0.3 cases per 100,000 amongst Japanese children [2]. Although the etiology of Behcet's disease is still unknown, studies have revealed an association between tumor necrosis factor-alpha (TNF-***α***) and the clinical features, and the efficacy of some anti-TNF agents is reported in Behcet's disease patients [3]. We report on a juvenile patient with corticosteroid-dependent intestinal Behcet's disease who responded to subcutaneous adalimumab injections.

## 2. Case Report

**Figure 1:**
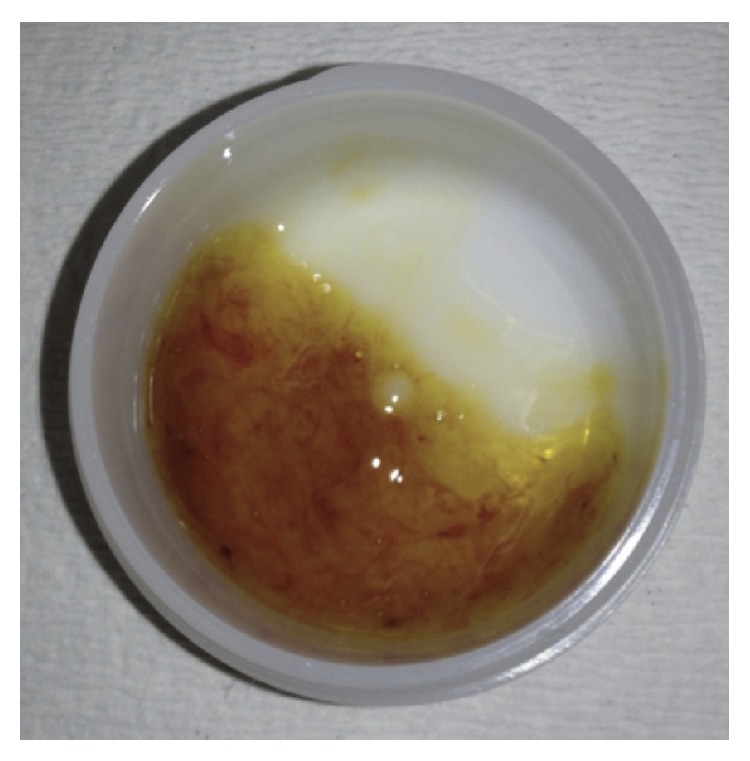
Hematochezia. The patient experienced abdominal pain with frequent hematochezia.

**Figure 2:**
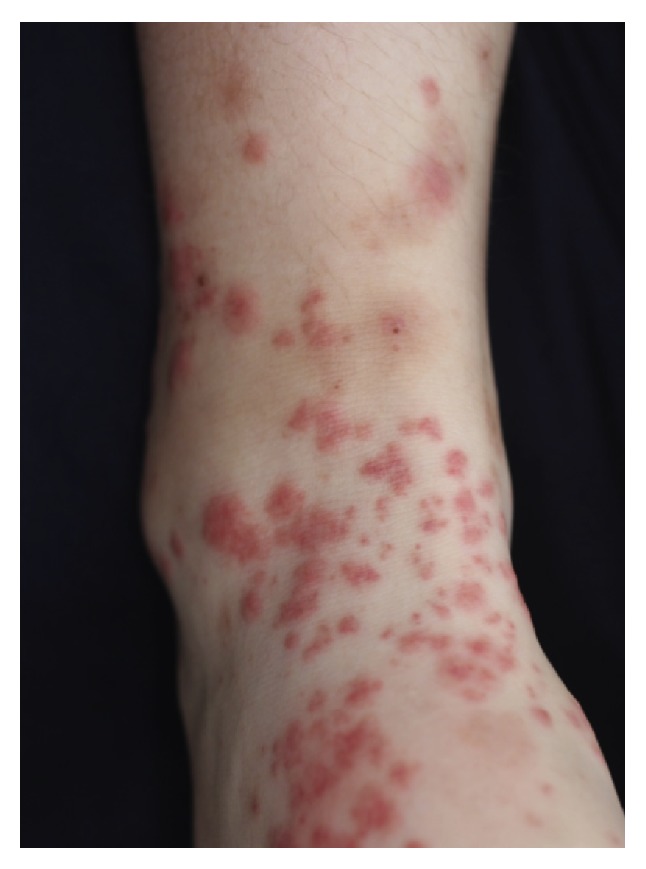
Skin lesions noted in the patient. The patient had edematous erythema and erythema-nodosum-like eruptions on the lower extremities.

**Figure 3:**
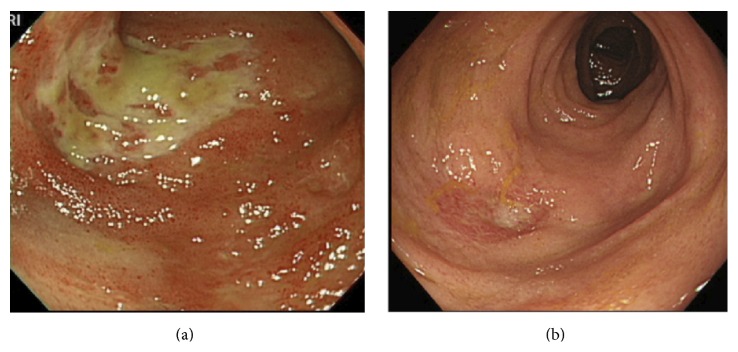
Lower gastrointestinal endoscopic observations. (a) On admission, lower gastrointestinal endoscopy revealed ulcers in the terminal ileum. (b) A week after the initiation of adalimumab, lower gastroendoscopy showed epithelialization of the ulcers.

The patient, a 13-year-old girl, had a second-generation Korean father, a Japanese mother, an elder brother, and a younger sister. There was no family history of autoimmune disease. The patient was admitted to our hospital for acute tonsillitis a year before the current admission. She had oral aphthosis recurring more than three times in 12-month period and repeated genital aphthosis. The patient sought evaluation at our hospital for oral ulcers, vulvar pain, headaches, arthralgias, and erythema on the lower extremities in June 2013. She was admitted to the dermatology department. Following the gradual onset of abdominal pain and bloody diarrhea (Figure 1), she was referred to the pediatric department. On admission, the patient had a temperature of 36.2°C, a heart rate of 81 beats per minute, and a blood pressure of 130/86 mmHg. At the time of admission, her physical findings were appropriate for her age, but she lost 2.2 kg during the first week while being admitted to the dermatology department. Edematous erythema and erythema-nodosum-like eruptions were noted on the lower extremities (Figure 2). There were painful ulcers on the left buccal mucosa and left labia minora. Blood testing revealed a significant elevation of the white blood cell count (14.17 × 10^3^/*μ*L) and C-reactive protein (CRP; 4.29 mg/dL), fibrinogen degradation products (FDP; 27.9 *μ*g/mL [nl < 4.0]), D-dimer (15.60 *μ*g/mL [nl < 1.0]), and IgD levels (27.5 mg/dL [nl < 9.0]). Human leucocyte antigen (HLA)-B51 was positive. An abdominal CT showed a thickened colonic wall. Lower gastrointestinal endoscopy revealed ulcers in the terminal ileum (Figure 3(a)) and biopsies of the ileum revealed nonspecific chronic inflammation. The ophthalmologic examination did not show the presence of uveitis. Pathergy testing was negative. Histology of the skin lesions on the lower extremities revealed neutrophilic dermatitis in the dermis. The symptoms met the criteria established by the International Study Group for the diagnosis of Behcet's disease [4, 5] with gastrointestinal manifestations.

**Figure 4:**
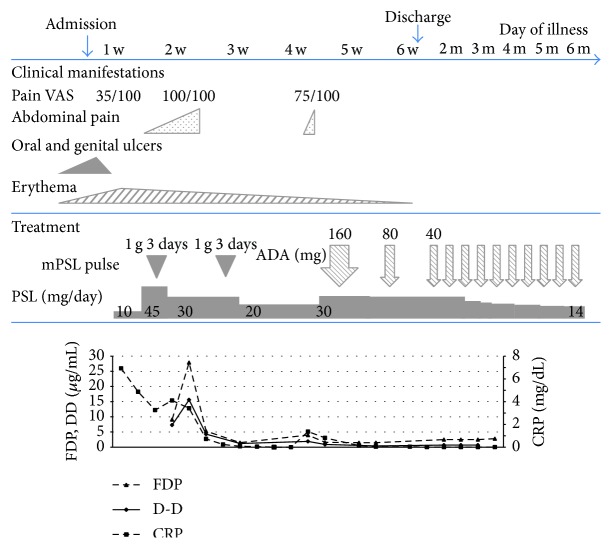
Clinical course of the patient. Corticosteroid pulse therapy with methylprednisolone was administered to the patient. After two courses of corticosteroid pulse therapy, the symptoms and blood test results showed improvement. The abdominal pain relapsed while tapering the dose of corticosteroid. The patient was begun on adalimumab at a dose of 160 mg in week 0, 80 mg in week 2, and 40 mg every other week thereafter. The clinical manifestations of Behcet's disease resolved soon after the initiation of adalimumab.

Fasting with total parental nutrition was indicated, and corticosteroid pulse therapy with methylprednisolone (1 g/day for 3 days) was initiated. After two courses of corticosteroid pulse therapy, the abdominal pain and hematochezia resolved. Blood testing showed improvement in inflammatory and fibrinolytic markers. One week after the dosage of corticosteroid was reduced to 20 mg/day, the abdominal pain recurred. Although there was no reactivation of oral and genital ulcers or exacerbation of the rashes, blood testing showed reelevation of CRP, FDP, and D-dimer levels. Because the clinical findings were corticosteroid-dependent, the patient was begun on adalimumab, a fully humanized anti-TNF-***α*** monoclonal antibody. She received subcutaneous injections of adalimumab at a dose of 160 mg in week 0, 80 mg in week 2, and 40 mg every other week thereafter. The symptoms and clinical manifestations resolved soon after the initiation of adalimumab. The circulating inflammatory markers converted to negative. Initiation of the adalimumab made it possible to reduce the corticosteroid dose without any symptoms (Figure 4). Epithelialization of the ulcers was demonstrated on lower gastrointestinal endoscopy, which was performed 1 week after the initiation of adalimumab (Figure 3(b)). Although the oral corticosteroid dose was gradually decreased, the symptoms never recurred. No adverse effects of adalimumab were observed.

## 3. Discussion

The first case in which adalimumab was used for pediatric gastrointestinal Behcet's disease has been reported.

Behcet's Disease Working Group of the Pediatric Rheumatoid Association of Japan reported that pediatric cases have more gastrointestinal manifestations (62% versus 23% in adults) and less uveitis (32% versus 61% in adults) than in adults. Because the frequency of clinical features between adults and children is different, approximately one-half of the pediatric cases have not satisfied international diagnostic criteria, and many juvenile patients have never been diagnosed.

The ulcers in patients with Behcet's disease and gastrointestinal involvement are most commonly found in the terminal ileum and cecum. Sometimes it is difficult to distinguish Behcet's disease with gastrointestinal involvement from inflammatory bowel diseases, such as Crohn's disease. It was reported that 16 of 43 adults with intestinal Behcet's disease underwent surgery because of acute abdomens and/or massive bleeding [6]. Moreover, a history of frequent relapses is a risk factor for intestinal stenosis; thus, it is essential to achieve remission.

The etiology of Behcet's disease is still unknown. The strongest genetic marker in Behcet's disease is HLAB^*∗*^5101 [7]. In healthy population of most ethnic origins, the frequency of HLA51 is around 20% whereas it is increased to 50% to 80% in Behcet's disease [7]. Recently, cytokines have been shown to play important roles in the immunopathogenesis of Behcet's disease [8].

Because there is no therapeutic strategy specific for pediatric cases of Behcet's disease, juvenile patients are treated using the standard adult regimen. However, there are no internationally accepted, standardized treatment strategies for gastrointestinal Behcet's disease so far. A report showed that 40% of adult intestinal Behcet's disease patients did not respond to corticosteroid treatment [4]. Intractable cases occasionally failed in intestinal perforations and gastrointestinal surgery may cause severe, extended pathergy phenomenon with lethal outcome [9]. The prolonged use of corticosteroids in pediatric patients is associated with growth disorders, abnormal bone metabolism, and mental disorders. For steroid-dependent or resistant cases, 5-ASA/sulfasalazine or azathioprine is considered second-line treatment (Table 1 [10]). The Japanese Inflammatory Bowel Disease Research Group proposed a set of consensus statements in 2007, updated again in 2014, to address growing low-level evidence supporting the use of anti-TNF therapy [11, 12]. Recently, many reports have shown the benefits of anti-TNF-***α*** agents in the treatment of Behcet's disease (Table 1 [10]). Although the mechanism of action is not completely clear, the following anti-TNF-***α*** agent findings are relevant: (1) neutralizing activity by binding to soluble/membrane-bound TNF-***α***; (2) inducing apoptosis by binding to membrane-bound TNF-***α*** on T cells or monocytes; and (3) regulating the immune system via production of anti-inflammatory cytokines, inhibiting T cell amplification, and binding to macrophages or Fc receptors.

In gastrointestinal Behcet's disease, infliximab and adalimumab are the two best studied biologic agents. Currently, etanercept has no role in the management of refractory intestinal Behcet's disease in the recent guideline by the Japanese Inflammatory Bowel Disease Research Group though one case report was reported. The first reports of infliximab success in patients with glucocorticoid dependent Behcet's disease were published in 2001 [26, 27]. A Korean multicenter retrospective study of infliximab with 28 intestinal Behcet's disease patients demonstrated that older age at diagnosis (>40 years), female sex, longer duration of disease (>5 years), concomitant immunomodulator use, and achievement of remission within 4 weeks were predictive of sustained response [22]. In Japan, infliximab was approved by Ministry of Health, Labour and Welfare for the treatment of uveitis in Behcet's disease in 2010 and gastrointestinal, neurological involvements and vasculitis in 2014. Adalimumab was the first fully human monoclonal antibody drug approved by Food and Drug Administration. It has a terminal half-life comparable to that of human IgG1 (approximately two weeks) and is available for home subcutaneous injection [28]; thus, adalimumab has distinct advantages for pediatric use. According to a molecular biological study, adalimumab occupies a larger area on the antigen-antibody interface and has a higher affinity than etanercept and infliximab [29]. The efficacy and safety of adalimumab were investigated in a prospective, nonplacebo controlled, multicenter trial in Japan of 20 patients (10 male and 10 female, 24 to 66 (42.4 ± 13,3) years of age) with refractory intestinal Behcet's disease and the dosing regimen of adalimumab was followed in this study [24]. Nine (45%) and 12 patients (60%) demonstrated improvement at 24 and 52 weeks, respectively. Four patients (20%) achieved complete early and late remission at weeks 24 and 52, respectively. Furthermore, 8 of 13 patients completely discontinue corticosteroids during the study. This case was not enrolled in the clinical trial but was able to be treated with adalimumab because it was approved for the treatment of intestinal Behcet's disease in May 2013 in Japan.

The safety of adalimumab in pediatric patients has already been discussed with respect to juvenile idiopathic arthritis [30]. It should be a good choice for patients with intestinal Behcet's disease refractory to conventional treatment though continued discussion about the safety and efficacy of adalimumab is expected.

## Figures and Tables

**Table 1:** Summary of the highest level of evidence for immunosuppressants and anti-TNF biologics [10].

Therapy	Level(s) of published evidence
5-ASA/sulfasalazine	Retrospective cohort study [13]
Corticosteroids	Expert opinion, European League Against Rheumatism Recommendations [14]
Thalidomide	Case reports [15–17]
Azathioprine, 6-MP	Retrospective cohort studies [13]
Mycophenolate	Case report [18]
Methotrexate	Case series [19]
Tacrolimus	Case report [20]
Infliximab	Single arm clinical trial [21]
	Retrospective cohort study [22]
	Case series [23]
Adalimumab	Prospective, nonplacebo controlled clinical trial [24]
Etanercept	Case report [25]

## Abbreviations

ADA: Adalimumab
CRP: C-reactive protein
D-D: D-dimer
FDP: Fibrinogen degradation products
mPSL: Methylprednisolone
PSL: Prednisolone.
